# Multi-century long density chronology of living and sub-fossil trees from Lake Schwarzensee, Austria

**DOI:** 10.1016/j.dendro.2014.11.004

**Published:** 2015

**Authors:** Marzena Kłusek, Thomas M. Melvin, Michael Grabner

**Affiliations:** a University of Natural Resources and Life Sciences Vienna, BOKU, Konrad Lorenz Straße 24, 3430 Tulln an der Donau, Austria; b Climatic Research Unit, University of East Anglia, Norwich NR4 7TJ, United Kingdom

**Keywords:** Density chronology, Maximum density, Light rings, Sub-fossil wood, Alps

## Abstract

This paper presents a multi-century, maximum latewood density (MXD) chronology developed from living and sub-fossil spruce trees from the Eastern Alps. The chronology is continuous from 88AD to 2008AD. This time series has been analysed with respect to its possible use for climate reconstruction. Correlations with climatic data showed strong dependence between MXD of growth rings and temperature of April, May, June, July, August and September and a weaker, negative dependence with precipitation of May and September. For solar radiation a positive relationship was noted for April, July, August and September. Light rings were frequently observed within the analysed samples and the climate of years with light rings was examined. Mean monthly temperatures in January, June, August, September and October, averaged during light ring years, were cooler than during years without light rings. Precipitation was also significantly reduced in March during light ring years. In turn, solar radiation during light ring years has significantly lowered values in February and August. The occurrence of light rings was often positively related to strong volcanic events.

## Introduction

Densitometric growth-ring studies were initiated in the second half of the last century (Polge, 1970, 1978). Since that time they have become a commonly applied method in dendroclimatology (Briffa et al., 2001, 2004; Collins et al., 2002). It results from the fact that density chronologies are characterised by several features that make them especially appropriate for dendroclimatological research within temperature limited environments. In comparison to the studies based on the measurements of annual tree-ring width (TRW), maximum latewood density (MXD) chronologies demonstrate significantly higher correlation coefficients with climatic parameters and this strong relationship refers to a longer period within a growing season (Briffa et al., 1992b, 1994, 1998c). The age-related biological trends in MXD measurements tend to have lower amplitude, relative to the common signal, than those of TRW. Moreover, MXD chronologies possess a higher proportion of high-frequency variance than do TRW chronologies and contain a greater fraction of their signal in the higher-frequency domain (Briffa et al., 2002a,b). Therefore, the advantage of MXD over TRW for climate reconstruction may not extend to frequencies beyond inter-annual to multi-decadal wavelengths. Nevertheless, the ring width data might present more low frequency variations than the climate itself because they have tendencies towards greater persistence and biological as well as environmental feedbacks. These biological feedbacks likely enhance also an autocorrelation level which is much higher for TRW in comparison with MXD parameter (Frank and Esper, 2005a,b).

Other beneficial factor which predisposes MXD chronologies towards paleoclimatic research is low between-tree variance. The common signal of MXD is also less dependent on the ecological factors of the site (Schweingruber and Briffa, 1996; Wilson and Luckman, 2003). It has been found that when MXD chronologies are averaged within quite large areas, the correlation coefficient with similarly averaged temperature increases. Because of this fact MXD chronologies are useful for research over a wide territorial range (Schweingruber et al., 1993; Waldner and Schweingruber, 1996). Accordingly, the analysis of densitometric data could deliver a picture of climatic conditions on a greater spatial scale compared to similar analysis using TRW measurements (Briffa et al., 2002a,b). Additionally, the relationships between MXD and climate factors tend to be common across different genera of conifer wood. This allows the use of various tree species during the development of regional chronologies (Schweingruber et al., 1988; Luckman and Wilson, 2005).

This paper presents the results of X-ray densitometric studies conducted with living and sub-fossil wood samples originating from a site localised within the Eastern Alps. The resulting MXD time series have been analysed with respect to the possible use for further dendroclimatological research. For this purpose the influence of climate factors on ring density was examined. The relationship with climate conditions was also tested by the application of light rings. These results will be used for the reconstruction of climate which will be presented in full elsewhere.

## Materials and methods

### Wood origin and preservation

**Fig. 1:**
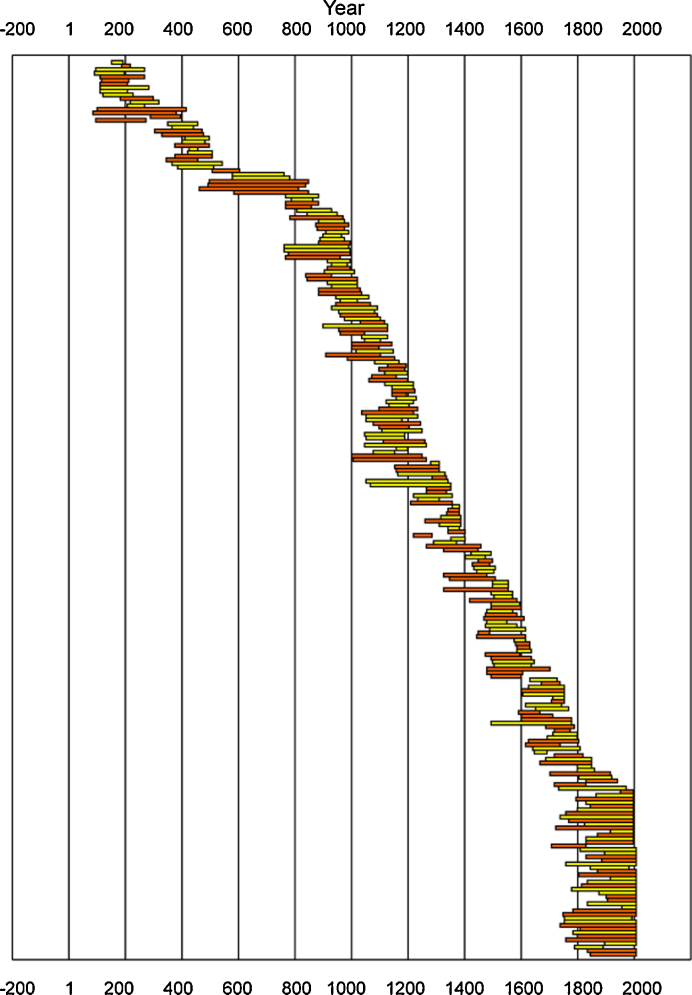
The time-spans of samples that constitute the Schwarzensee MXD chronology. Trees are sorted by their final year, all radii of a tree are the same shade and are adjacent, and alternate trees are darker or lighter.

The wood samples come from an area situated around a small mountain lake – Schwarzensee (47°31′N, 13°49′E, 1450 m.a.s.l.). This lake lies in the region of the Dachstein Mountains which belong to the Limestone Alps. The studied material consists of 50 living and 237 sub-fossil samples of Norway spruce (*Picea abies* (L.) Karst.). In total, 287 samples (radii) from 155 trees were used in this densiometric research. Usually two radii were measured for each tree (Fig. 1). Sub-fossil wood was collected as stem disks from trunks deposited on the bottom of the lake, all of which originated from trees growing on the nearby hills. These trees after the death slid down the steep slopes and cliffs and finally sank into the water. The recent samples were cores extracted from the trees growing around the lake. The stands of living trees were situated approximately within a 300 m radius. As a result, all samples come from a spatially very limited area. An overwhelming majority of the measured samples were derived from a previously constructed TRW chronology (Grabner et al., 2007). However, 20 living trees were sampled especially for this density analysis in the year 2009.

The storage under water allowed wood preservation in a relatively unchanged state. Limited oxygen access slowed down the development of aerobic bacteria and fungi that, under usual open-air conditions, lead to fast wood decomposition (Fengel, 1991). However, the degradation of the material was not completely prevented in this waterlogged environment because the wood was influenced by its penetration by mineral substances and organic matter dissolved in the lake water, and also by decomposition resulting from the activity of anaerobic microorganisms (Holt and Jones, 1983). These factors, acting over hundreds of years in the sedimentation environment, partially modified the chemical composition and physical properties of the wood (Kim and Singh, 2000). It was observed that destructive processes mainly encompassed the external zone of the trunks.

### Sample preparation

Narrow laths (approximately 1 cm × 1 cm), running from the pith to the external part of the trunk, were cut from the sub-fossil stem sections. These laths and the cores bored from living trees were glued onto wooden supports and then their tracheid direction was determined with a protractor. This measurement enabled the identification of the wood surface which at every point was perpendicular to the longer axis of stem. Next, thin strips (of thickness 1.2 mm) were cut in this perpendicular direction using a double bladed circular saw.

After cutting, the thin strips were rinsed in distilled water. For density analysis it is necessary to remove resins, heartwood substances and other organic and inorganic ingredients from wood (Grabner et al., 2005). These compounds are characterised by different X-ray adsorption rates in comparison with cell walls and for this reason they could bias the results of density measurements. In the process of extracting these compounds acetone, benzene or ethylene, and hot water are usually applied (Schweingruber et al., 1978; Grabner et al., 2005). However, the standard procedure was established for wood derived from living trees and it was necessary to try out various methods here to establish their usefulness in sub-fossil sample preparation. In the initial project phase, several substances were tested for their suitability. The optimal result was received by rinsing the sub-fossil and recent samples in distilled water, carried out for 24 h at room temperature. This was the best solution to remove unwanted organic and inorganic water soluble compounds without destroying the structure of wood. Subsequently, the samples were subjected to acclimatisation in order to produce wood with a uniform humidity. The moisture content of cell walls should be kept at constant level because its variability could result in invalid density measurements (Lenz et al., 1976). Conditioning was performed over a 48-h time span at a constant temperature of 20 °C and relative air humidity of 65%.

### Density measurements

After finishing the preparation process the samples were irradiated. For this purpose the strips were placed on an X-ray sensitive film together with a calibration wedge of cellulose acetate. Six steps of thickness were used for the calibration of wood density. Exposure of X-raying was performed from a distance of 2.5 m, during a 25-min period, with the device's accelerating tension of 24 kV and intensity of 10 mA, with application of Seifert ISO-DEBYEFLEX 3003 apparatus. Next, irradiated films were developed using a standard procedure and then digitalised by the means of a dendrodensitometer which was constructed at BOKU University. The X-ray densitometer was calibrated against the cellulose acetate wedges to develop a standard optical reference. Tree-ring transparency (grey level) was obtained at 5 μm intervals along the radial direction of the stem and from these the density of samples was determined using a computer programme compiled at BOKU University.

During measurement, degraded parts of the samples with lowered wood density were avoided. Resin canals, damages and cracks as well as the places where the sample surface was not sufficiently parallel to the cross-section of the stem were also omitted. Furthermore, zones of strongly developed reaction or juvenile wood were not used because the wood density within those special tissues poorly reflects the impact of climate (Lamy et al., 2012). At the time of analyses the presence of light rings and other wood structure disturbances were noted. As a result of the measurements, the values of maximum, mean and minimum wood density for each growth ring were produced. The average density of earlywood and latewood zones, the width of earlywood and latewood and the percentage of latewood in the growth ring were also calculated. The density boundary between earlywood and latewood was defined as the position of half way between the maximum and minimum values within an annual density profile (Lenz et al., 1976).

### Chronology construction

The width of growth rings (TRW) defined by densitometry were compared to the earlier dendrochronological data obtained for the same trees during previous TRW chronology development (Grabner et al., 2007). The determination of calendar dates of tree-ring formation was carried out by cross dating with the pre-existing ring-width master chronology. In this way the time series from the density measurements were linked to their corresponding years and an exactly-dated MXD chronology was built. The quality of the chronology crossdating was verified with the help of COFECHA (Holmes, 1983). The CRUST program was used (Melvin and Briffa, 2014) to derive tree indices and chronologies as described below. During chronology construction, to reduce within tree noise, where multiple ring measurements were available for a tree these were averaged together to produce mean-tree measurement series.

Individual series of measurements contain both the common climate signal and the age-related growth trend. These undesirable long-term variations are not climate dependent but arise from physiological ageing processes. In MXD series, after an initial juvenile growth period, there is a tendency for the MXD of rings to reduce as the age of the tree increases (Wilson and Luckman, 2003; Wimmer and Downes, 2003). In order to isolate the common climate-related signal these biological trends need to be removed (Cook, 1987; Cook et al., 1990).

**Fig. 2:**
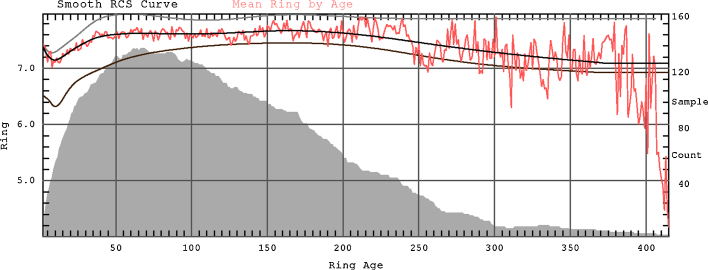
The RCS curves that were used for standardisation. Red line is the mean MXD by ring age, and black line is the count-weighted mean of the smoothed RCS curves. The two individual RCS curves, smoothed with age dependant spline, used for trees with higher and lower mean MXD are shown as grey and brown lines. The grey area shows mean-tree sample depth. (For interpretation of the references to color in this figure legend, the reader is referred to the web version of the article.)

**Fig. 3:**
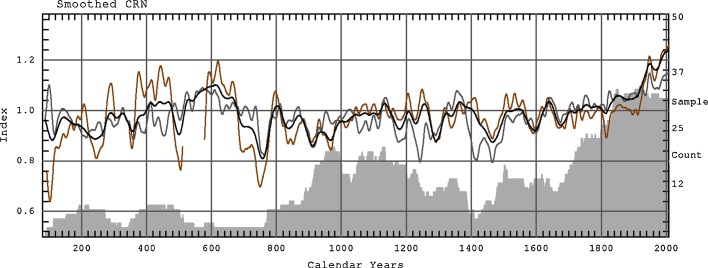
The sub-chronologies obtained from the two groups of higher and lower mean MXD are shown as grey and brown lines. Chronologies were smoothed using spline with 50-year frequency cutoff. Grey area show mean-tree sample depth. (For interpretation of the references to color in this figure legend, the reader is referred to the web version of the article.)

In the Regional Curve Standardisation method (RCS) the mean growth trend is estimated and removed from each tree series. This method is used here. During RCS curve preparation particular series of measurements were aligned by ring age and averaged. Because measurement series come from a wide range of times during this procedure the amplitude of the common signal was reduced while the ‘average’ age-related-growth-trend was preserved (Briffa et al., 1992a; Esper et al., 2003). Moreover, to remove the common signal prior to creating RCS curves the signal free method with maximum 10 iterations was applied for individual measurement series (Melvin and Briffa, 2008). In order to reduce the potential for systematic bias (Melvin et al., 2013), here two-curve RCS was used to standardise the mean-tree MXD chronology. For this purpose the data were split into two equal groups according to higher and lower mean MXD and each group was standardised with its own RCS curve (Figs. 2 and 3). RCS curves were smoothed with an age dependant spline (Melvin et al., 2007). Indexes were calculated as ratios and the chronology was computed as the arithmetic mean. Variance stabilisation of the chronology was performed using Keith Briffa's RBAR- weighted method (Osborn et al., 1997). The resultant standard chronology was analysed with respect to the potential for its application to dendroclimatological research.

### Correlations with climate parameters

The MXD chronology was correlated with gridded temperature and precipitation time series for the area of the lake (Auer et al., 2005; Efthymiadis et al., 2006; Chimani et al., 2013) and data from the 1/6th of a degree grid box centred at 47°35′ N, 13°45′ E was used. Measurements of solar radiation originated from the meteorological station of Kremsmünster (48°06′ N, 14°13′ E, 389 m.a.s.l.) (Auer et al., 2007). However, this radiation data should be treated with caution because Kremsmünster is 96 km from Schwarzensee and is situated at a much lower altitude. The insolation level measured for this lowland and distant station may differ considerably from the real situation in a mountain area. In total, the meteorological records encompassed monthly averaged data for temperature (1780–2008) and for solar radiation (1884–2008), as well as monthly sums of precipitation (1800–2008).

The relationship between chronology and climate conditions was determined on the basis of correlation coefficients and response function values. In each case, only one instrumental parameter was included in the response function analysis and all time intervals from the previous August to October of the current year were tested. Moreover, an additional response function analysis that encompassed both temperature and solar radiation data was performed. For better comparison, analyses were undertaken over a common period 1884–2008. The calculations were carried out using the DENDROCLIM 2002 program (Biondi and Waikul, 2004).

In the next stage of research the connection between climate and light rings was examined. These rings possess a latewood zone with thin-walled cells and are also marked by lower MXD values (Filion et al., 1986; Liang et al., 1997; De Grandpré et al., 2011). In this study light rings were specified by means of two methods. They were identified visually on the basis of the dark colour of latewood visible on an X-ray sensitive film. In this case, light-ring years were classified as those years when at least 5% of trees were characterised by the apparent decline of latewood density. This is the commonly threshold used in dendrochronology (Szeicz, 1996; Girardin et al., 2009). Light rings were also determined using maximum density measurements. For this purpose the mean value and standard deviation were calculated for each MXD series. If the maximum density of a ring was lower than two standard deviations below the series mean, then this ring was assigned to be a light ring (Wang et al., 2000). In this method a light ring year was defined to be a year for which at least 25% of samples demonstrated such strongly reduced MXD values.

With the aim of finding a relation between light rings and climate the meteorological measurements of temperature, precipitation and radiation were separated into two groups. One of them consisted of light ring years and the second was composed of remaining normal years. The Student-*t* test was utilised to verify if the difference between the mean values of climatic measurements obtained for these two groups was significant. This method had previously been applied to light rings analysis (Yamaguchi et al., 1993). Student-*t* test was calculated separately for visually distinguished and MXD designated light ring years.

Additionally, the occurrence of light rings years was compared with known volcanic activity. The co-incident development of light rings after volcanic eruptions has been previously noted many times (Filion et al., 1986; D’Arrigo et al., 2001). During the research here only the eruptions characterised by an explosivity index (Siebert and Simkin, 2002) equal to or greater than 4 were taken into consideration. The effects of such severe eruptions can be seen over a large territory, even at global scale and environmental changes arising as their result are observed not only for a single growing season but also for a few years after the event (Scuderi, 1990). Therefore, volcanoes located both in close and distant places were analysed and lags of up to 3 years were taken into account.

## Results

### Chronology characteristics

On the basis of densitometric research a new MXD chronology spanning the period from 889BC to 2008AD was created. This chronology, however, has gaps in this time period between 672BC and 599BC and between 37AD and 88AD. Overall, the time span of the continuous MXD chronology (88AD–2008AD) is much shorter than that of the pre-existing ring-width master chronology which was composed of the initial material (958BC–2008AD). Observed reduction of time-scale range is related to the state of wood preservation required for density measurement. As previously mentioned, some samples had deteriorated zones, characterised by decreased wood density. These fragments were especially numerous in the external part of the logs. Moreover, some samples possessed zones of strongly developed reaction or juvenile wood. All these parts were omitted during the densitometric research. And these samples with less than twenty measured growth-rings had been excluded from further research. As a result the density chronology consist of fewer rings from a smaller number of specimens than the initial ring-width master chronology.

The whole MXD chronology was composed of 264 spruce samples (Fig. 1). The number of growth rings in particular specimens varied from 26 to 354. The application of short series was possible because the measured strips were parts of previously dated stem discs. The mean segment length (average number of rings per core or disc) was 136 years and the mean length of segments in any year (average length of the samples occurring in a given year) reached at least 100 years during the continuous chronology period. Simultaneously, the mean inter-series correlation calculated using CRUST software amounted to 0.54.

**Fig. 4:**
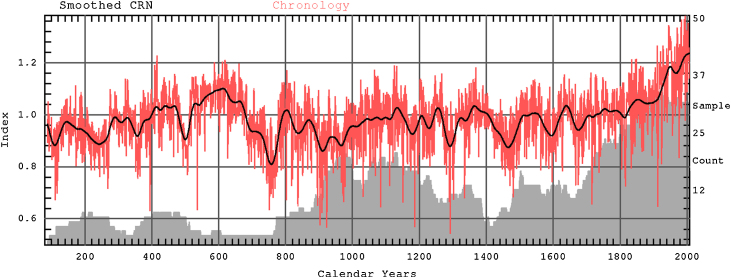
Schwarzensee MXD chronology (red line) and chronology smoothed using spline with 50-year frequency cutoff (black line). Grey area show mean-tree sample depth. (For interpretation of the references to color in this figure legend, the reader is referred to the web version of the article.)

The final MXD chronology demonstrated distinct long-term trends (Fig. 4). Positive growth tendencies were visible especially within the time range of about 400–670AD. This time span was broken by short-term growth decrease about 500AD. Higher MXD values were observed also about 1120–1250AD, 1300–1420AD and 1640AD. Nevertheless, the period 670–1800AD was characterised by rather low MXD data with only momentary fluctuations towards the increase of growth. The largest index values were expressed during the twentieth century. They reached maximum in years 1983AD, 1992AD, 1994AD and 2003AD. The growth increase began ∼1820 and climbed steeply since ∼1900AD. In turn, time intervals marked by strongly diminished growth covered the years 680–780AD, 880–980AD, 1270–1310AD, 1420–1500AD. The lowest MXD values occurred in years 903AD, 909AD, 923AD, 1186AD and 1292AD.

### Climate signal

**Fig. 5:**
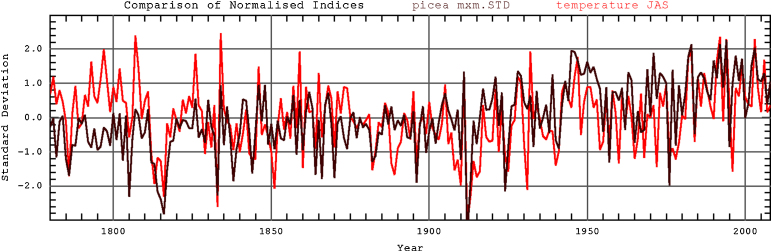
Comparison of mean temperature averaged for July, August and September (red line) with MXD Schwarzensee RCS chronology (brown line). (For interpretation of the references to color in this figure legend, the reader is referred to the web version of the article.)

The correlation of the MXD chronology with climate parameters is shown in Table 1. The best results were observed for temperature of July, August and September (Fig. 5) but also significant with April, May and June of the current year. In contrast to temperature, significant negative dependence was obtained for precipitation in May and September of the current year. Positive response to precipitation existed between November of the previous year and MXD. For solar radiation a positive relation with MXD was noted for April, July, August and September of the current year.

In comparison with maximum density, light rings were characterised by weaker relationship with climate. During conducted research 140 light rings were defined visually and 994 light rings were quantified. For the continuous chronology period 34 visually determined and 34 MXD calculated light ring years occurred, from which respectively 6 and 3 existed within the time span of the meteorological data. Light ring years distinguished on the basis of each of these two methods differed considerably especially in the poorly replicated parts of the chronology. Comparison of these years is presented in the Table 2. Therefore, to avoid mixing both types of light rings and to retain the homogeneity of an analysis as well as to be more consistent and comparable with other studies published so far only the 6 visual light ring years were incorporated into further research.

To establish the climate signal of light rings the monthly values of meteorological parameters were averaged separately for light-ring years and for remaining normal years within the time span (1780–2008AD) of the climate data and the results were analysed using the Student-*t* test (Table 3). It was found that mean monthly temperatures during light ring years were cooler than during remaining years for all months. However, temperature differences were significant only in January, June, August, September and October and significance level reached the highest value in August. Differences in precipitation were significant for March and for this month light rings years were characterised by lower snowfall than remaining years. In turn, solar radiation during light ring years had significantly reduced values in February and August.

The occurrence of light rings was also compared with strong volcanic events. The relationship between those variables was rather ambiguous, however, within the more contemporary part of the chronology a coincidence among them could be observed. For the initial part of chronology the comparison between light ring years and volcanic activity was saddled with a large uncertainty which arises from the imprecise dating of the oldest eruptions. Nevertheless, within the last 500 years the convergence between light ring and volcanic cooling was high. Among 9 light ring years distinguished during this period only 2 of them were not associated with volcanoes. The origin of remaining 7 light rings was related to volcanic forcing. Although it is rather difficult to unambiguously ascribe all these light rings to a particular eruptive event however, most probably light rings which appeared in years 1675, 1716 were triggered by the eruption of Gamkonora (1673 May 20) and Chirpoi (1712 December 31 ± 365 days) respectively. Light ring years 1814 and 1816 could be paired with Soufriere St. Vincent (1812 April 27), Awu (1812 August 6), Suwanose-Jima (1813), Mayon (1814 February 1) or Tambora (1815 April 10) impact. In turn, light ring year 1912 could be linked with Lolobau (1911), and Novarupta (1912 June 6) activity, whereas light ring year 1924 coincides with Raikoke (1924 February 15) eruption, and light ring 1976 could result from Tiatia (1973 July 14), Fuego (1974 October 17), Tolbachik (1975 July 6) or Augustine (1976 January 22) influence. On the other hand, it should be stressed that some strong volcanic events did not cause light ring development for the Schwarzensee chronology. Among three largest eruptions in the last two millennia that occurred around AD 536, 934 and 1258 (D’Arrigo et al., 2001) only the first of them was reflected in Schwarzensee light rings.

## Discussion

From the Alps more than 50 maximum density chronologies have been developed so far. They were prepared for various coniferous species. Many of these chronologies were analysed as a component of much larger dendrochronological networks (Briffa et al., 2001, 2002a,b; Collins et al., 2002). However, only three Alpine chronologies cover time period longer than one thousand years. Among them, the nearest to Schwarzensee in the terms of distance and similarity is MXD chronology from Tyrol. Samples combined in the Tyrol chronology were derived from two neighbouring valleys which are situated south of Innsbruck in western Austria. This chronology integrates measurement series from living trees and from historic wood. It ranges from 1053AD to 2003AD and is composed of Norway spruce (*Picea abies* (L.) Karst.) (Esper et al., 2007).

**Fig. 6:**
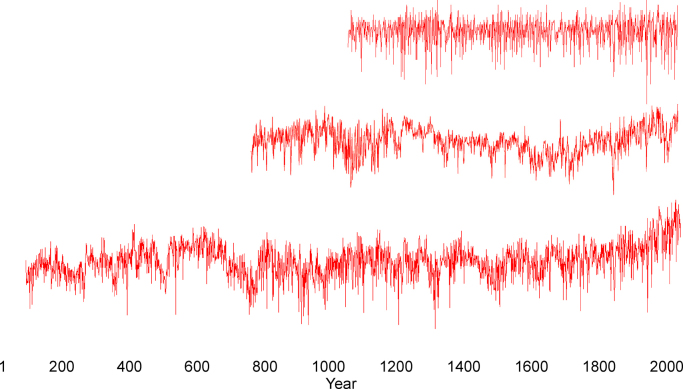
Comparison of the new chronology from this work (bottom) with Lötschental (middle) and Tyrol (uppermost) chronologies.

In spite of the many similarities Tyrol chronology is not identical to Schwarzensee ones. The main differences are manifested in the weak compatibility of long-term trends. Tyrol chronology demonstrates positive growth tendencies within the period about 1200–1300AD, 1450–1550AD and 1930–1970AD. In turn, low MXD values occur within the time span about 1100–1200AD, 1300–1500AD and 1600–1800AD (Fig. 6). Nevertheless, all these low frequency variations are poorly marked and they are difficult to recognise since Tyrol time series contains a great fraction of its signal in the higher frequency domain.

The second long MXD chronology, which was developed in an area relatively local to Schwarzensee, is the larch (*Larix decidua* Mill.) Lötschental chronology from Swiss Alps (Büntgen et al., 2006). It reaches back to the 755AD. This chronology was constructed on the basis of wood samples derived from near timberline sites and wooden elements taken from subalpine buildings. Analysis of long-term trends observed within this Swiss chronology indicates positive growth tendency in the tenth and thirteenth century that correspond with twentieth century conditions. On the other hand, this time series displays a prolonged trend reduction from 1350AD to 1700AD. Swiss chronology demonstrates high MXD values in ∼970AD, ∼1150AD, ∼1230AD, and ∼1940AD, and during the most recent decade of chronology, with 2003AD reaching the highest index value since 755AD. Within the early period of high index values, a distinct depression occurs during the eleventh century. A long period of MXD decrease exists from ∼1350AD to 1820AD, with low MXD data in ∼1460AD, ∼1590AD, ∼1680AD, and ∼1820AD, and with 1816AD showing the lowest value over the past 1250 years (Büntgen et al., 2006) (Fig. 6). These characteristics distinguish Swiss chronology from Schwarzensee. The observed difference in the low frequency domain may be a result of the place of wood origin since in mountain regions a wide diversity of climate conditions occurs even at a close distance but also it could arise from the large error margins on low-frequency variance that are often present in MXD chronologies.

Despite many divergences between long-term trends all Alpine MXD chronologies are marked by strong similarity in their high-frequency response to climatic factors. The analyses conducted for the Schwarzensee chronology showed significant connections between the measured ring parameters and weather conditions. The observed high correlation between MXD and meteorological data can be largely explained by plant physiology because besides the genetically determined characteristics of individual species, wood density depends on temperature, radiation and water availability. Weather variables have an impact on cell turgor as well as on the level of hormonal and nutrient substance's production of a tree. They have indirect effects on photosynthesis, respiration, transpiration and related processes. In this way climate induces the changes in size and wall thickness of newly-created wood cells (Kozlowski et al., 1991; Von Wilpert, 1991; Wimmer, 1995). During the first part of the growing season climatic factors exert their strongest influence on radial enlargement of cells, whereas during the later part of the vegetation period, weather parameters mainly affect the cell wall thickening process (Rossi et al., 2006a,b).

Here we have shown that the highest correlation exists between maximum latewood density and temperature of late summer months. The best relationship was obtained for July, August and September. Taking wood anatomy into account, maximum latewood density is more dependent on the cell wall thickness than on cell size in the latewood (Wimmer and Grabner, 2000). In turn, the main factor, controlling the thickness of cell walls, is the duration of the cell wall thickening phase (Yasue et al., 2000). In northern and Alpine regions, the time span of the maturation of latewood cells is mainly conditioned by summer month's temperature and the time of cessation of the growing season. Also the length of the vegetation period is mainly influenced by thermal conditions. Therefore, the maximum density of latewood successfully reflects the temperatures of late summer months of these regions (Schweingruber et al., 1993; Wimmer and Grabner, 2000; Davi et al., 2003; Sano et al., 2005; Tuovinen, 2005).

In comparison with temperature, the connection between maximum density and precipitation is rather ambiguous. For the chronology from Schwarzensee a negative relationship occurred between maximum density and rainfall for spring and summer months. Such interdependence was observed for May and September of the current year. Inverse correlations with spring precipitation were seen previously at numerous Alpine sites and they are perhaps related to the delay in the onset of the growing season due to late winter snow (Frank and Esper, 2005b). In turn, the negative correlation between maximum density and summer rainfall might be an effect of high moisture content during cell enlargement phase. Under these circumstances water moves into the vacuoles, turgor grows, cell wall stretches and as a result lumen area increases (Rossi et al., 2006a). Moreover, cell size is also positively influenced by the amount of hormones produced by the young needles and consequently directly proportional to rainfall (Wimmer, 1995). In these two ways, high precipitation leads to wider tracheids formation and causes a reduction of the maximum density of wood. The interaction between maximum density and rainfall could be also explained by an opposing tendency which exists between high level of soil water and the cell maturation rate. This may in part be an effect of decreasing aeration in saturated soil and may be attributable to lowered crown activity, weakened root growth or other physiological responses occurring under such growth limiting conditions (Horacek et al., 1999). Furthermore, the negative correlation obtained for September month could be also the result of inverse co-variation of summer temperature and precipitations in the Alps (Frank and Esper, 2005a). Such phenomenon follows from diminished temperature and sunshine values of rainy days (Yasue et al., 2000).

The insolation level affects the density of wood because the smaller amount of incoming solar radiation could reduce the photosynthesis rate (Gindl, 1999; Berninger et al., 2004). The cell wall thickness is enlarged mainly by the addition of the secondary walls and therefore, is positively related to assimilate delivery (Liang et al., 1997). This explanation is also supported by results received during this research since for Schwarzensee MXD chronology a positive correlation with solar radiation was noted for April, July, August and September months.

Similar dependences as in the case of the MXD exist between climate and the occurrence of light rings. This compatibility is justified by the fact that these rings are characterised by a decrease in the density and the lignification level of the latewood cells. Light rings are bright-coloured because the latewood cell walls are not as thick as those of usual rings (Yamaguchi et al., 1993; Tardif et al., 2011). Thinner cell walls of latewood play a decisive role in light rings identification. However, these rings also possess significantly lower latewood cell number in comparison with normal ones. Light rings tend to have fewer earlywood cells as well (Volney and Mallett, 1992; Wang et al., 2002).

In Alpine regions, the appearance of light rings results mainly from the impact of low temperatures. Disadvantageous thermal conditions of the environment could inhibit each step of tree-ring formation, because they influence physiological processes of trees. Low temperature could reduce photosynthesis rate and net assimilation, could also slow down cell division and shorten the period of latewood growth, tracheid maturation and cell wall thickening phase. Therefore, diminished temperature leads to a production of fewer cells and to a formation of smaller cell diameters as well as to an incomplete latewood cell wall development (Liang et al., 1997). Light rings may arise as an effect of decreasing the temperature before the start of the vegetation period, during its duration or at the end of the growing season (Wang et al., 2000). In the study area the shortened length of the growing season and the occurrence of an early-ending autumn increase the chance of light ring appearance to the greatest extent (Gindl, 1999). However, in the case of the Schwarzensee MXD chronology temperature during light ring years was significantly cooler in comparison with normal years not only during autumn months but in January, June, August, September and October as well.

Therefore, it could be stated that for the analysed chronology the relationship between temperature and light rings is clearly visible (Table 3). Nevertheless, in many cases the frequency of light rings does not correlate unambiguously with corresponding temperatures averaged for particular months (Wang et al., 2000). Some light rings occur in fairly warm years, whereas frequently they can’t be found in rather cold ones. This indicates that light ring genesis may be linked with an intense short-term events. Hence, if light rings are found in several trees within a region they could be used as bio-indicator for extreme environmental factors (Hantemirov et al., 2004; Liang and Eckstein, 2006). The strong impact of extreme weather conditions on the production of light rings may also explain common lack of correlation between ring-width and light rings (Szeicz, 1996; Gindl, 1999; Hantemirov et al., 2004).

Besides temperature the development of light rings could be in some degree, the result of reduced solar radiation because incoming sunlight influences the level of the photosynthesis rate. This is confirmed by significantly lower values of solar radiation in February and August observed during Schwarzensee light ring years. However, most probably the combined effect of decreased temperature and diminished insolation might be the reason for light ring formation in timberline spruce (Gindl, 1999; Gindl et al., 2001).

Lowering the temperature and limiting the inflow of sunlight also occur as a consequence of volcanic activity. During intense eruptions large amounts of volcanic gases, aerosols and dusts are released into the atmosphere on hemispheric and global scales. This leads to drop in air temperature, an increase in relative humidity and a possible reduction in photosynthetic active radiation (Briffa et al., 1998b; Battipaglia et al., 2007; D’Arrigo et al., 2013). Volcanic eruptions may therefore contribute to the development of light rings (Sadler and Grattan, 1999; Biondi et al., 2003). An example of such a situation could be the year 1912. In this year light rings were formed in conifers in large parts of Europe. This year was characterised by particularly adverse growing conditions in Austria: from August to October the monthly mean temperatures were well below the long term mean, and September was the coldest in the history of instrumental temperature records in Austria. These outstanding meteorological conditions were at least in part caused by the eruption of Novarupta in south-western Alaska in June 1912 (Gindl and Grabner, 2000).

Observed interactions do not preclude the fact that some effect on the creation of light rings may be also exerted by moisture availability. For the Schwarzensee chronology the difference in precipitation between light ring years and normal years was significant for March and for this month light rings years were characterised by lower snowfall than remaining years. This points to water scarcity as a cause of light ring development. In dry area moisture deficit can lead to closure of the stomata, and also to some needle shedding or even to damage of the chloroplasts. The direct consequence of these processes is a reduction of the photosynthetic rate and the occurrence of light rings (Liang and Eckstein, 2006). Despite the fact that drought is not a major problem in the area of this research, water deficiency may, to some degree, have contributed towards the formation of the observed light rings. Such phenomenon could explain numerous light rings appearing in 1976. In this year a strong drought took place in Europe and simultaneously, August and September were characterised by low temperature most likely caused by an eruption of Tiatia (1973 July 14), Fuego (1974 October 17), Tolbachik (1975 July 6) and Augustine (1976 January 22) volcanoes. Therefore, in this year light rings could arise as a result of combined action of moisture deficit, cold late-summer temperatures and decreased level of solar radiation. Nevertheless, in the Alps, the main cause of light rings, as well as a main reason for diminished density of wood, is the impact of summer temperature (Gindl, 1999; Gindl et al., 2000). Unfavourable thermal conditions are the most important factor limiting the growth of trees in this region. Temperature influences the length of vegetation period and its value does not reach the optimum suitable for the plants’ biological processes (Leal et al., 2007).

A significant role of temperature is the main factor due to which the Schwarzensee chronology exhibits analogies with other Alpine chronologies. The resemblances between above described Tyrol chronology and Schwarzensee manifest among other things in the strong connection that for both exists between latewood maximum density measurements and August–September mean temperature values (Esper et al., 2007). The larch chronology from Swiss Alps also has a similar climate response to that of Schwarzensee. For Swiss chronology strong dependence between MXD and temperature occurs particularly in June, July, August and September. Simultaneously, this chronology is marked by weak, negative linkage with precipitation for summer months and by lack of any significant correlation with temperatures of previous year (Büntgen et al., 2006). All of these statistics are in accordance with climate signal obtained for Schwarzensee.

The same conclusions could be also drawn from analysis of dendrochronological network derived from Switzerland, France, Italy and Austria territories (Frank and Esper, 2005b). MXD chronologies which are part of this network demonstrate positive correlations with temperatures during April, May and July with peak values in August and September, as noted here. Within this Alpine dataset numerous sites are characterised also by negative relationship with summer precipitations likewise, as in the case of Schwarzensee. On the other hand, the negative impact of current February rainfall is usually observed for these chronologies (Frank and Esper, 2005b) while for Schwarzensee no significant values occur for this month. In contrast to Schwarzensee some chronologies included into the network reveal dependence on temperature of the prior season and especially positive influence of previous March (Frank and Esper, 2005b). Such connection was not noticed for Schwarzensee where no association exists between maximum density and temperature of previous year. Small discrepancies that appear between Schwarzensee and other Alpine MXD chronologies are attributable to differences in chronology construction techniques. However, most probably they result mainly from diverse geographical location of sites.

The tendencies towards greater incompatibility in climate signal between Schwarzensee and other MXD Alpine chronologies intensify along with increased inter-site distance and are amplified also by altitude changes. An example of such a situation could be the MXD chronology from south-eastern Alps which was composed of Norway spruce growing at a typical lowland plantation and at natural Alpine stand. Detailed analysis demonstrated that correlation coefficients calculated between this chronology and climate variables are consistent for both of these localities and revealed that although maximum latewood density is influenced by temperature of summer months however, only September has significant values of this coefficient. The relationship is positive for temperature and negative for precipitation dataset. Therefore, it could be inferred that in comparison with other Alpine stands the south-eastern Alps are marked by strong reduction in sensitivity level in relation to weather conditions (Levanič et al., 2009).

## Conclusion

Our study has shown the existence of high correlation between the measured tree-ring parameters and climatic factors. This is a very promising result, because Schwarzensee chronology has many features that predispose it to paleoclimatic research. The first aspect is the location of the site in the Alps almost at an elevation of regional timberline. This territory is characterised by cold temperatures and high snow cover. In this area temperature is the main factor that restricts tree growth and influences the length of the vegetation period. It predestines the chronology for the reconstruction of thermal conditions of environment. The next reason that promotes climatic studies is the very small territorial range of this chronology. All the trees come from the mountain slopes surrounding the small Schwarzensee lake. It secures climatic homogeneity of the site and also ensures small variability in sensitivity and in climate-growth relationship across the trees. Another beneficial factor existing for this chronology is the long-term meteorological record available in this area, dating back to the eighteenth century (Böhm et al., 2001). Access to meteorological data encompassing a period of more than two hundred years increases the precision of calibration and verification procedures which are carried out during dendroclimatic research. Application of climatic data reaching back to the pre-industrial period also allows the reduction of uncertainties. Such uncertainties are an effect of an anthropogenic influence. The dependence between maximal ring density and temperature can be distorted by air pollution or by an increase in UV radiation which is in turn caused by a decrease in concentration of atmospheric ozone. These factors reduce ring width, latewood proportion and density of wood. On the other hand, changing air CO_2_ concentration, fertilisation by nitrogen deposition from air pollution by NO_2_, and forest management practices, can stimulate productivity of trees (Sander et al., 1995; Dünisch et al., 1996; Kim and Fukazawa, 1997). All these factors can influence the robustness of climate reconstructions. Therefore, the calibration of chronologies against meteorological data from the pre-industrial time can minimise recent bias resulting from anthropogenically induced tree-ring density changes (Briffa et al., 1998a, 2004; Rolland et al., 1998; Wimmer and Downes, 2003).

## Figures and Tables

**Table 1:** Bootstrap correlation and response function values calculated in DENDROCLIM 2002 between mean monthly temperature, precipitation and radiation, and Schwarzensee MXD chronology. Significant values at the 0.05 levels are marked in bold. Asterisks indicate the year preceding growth-ring formation. Analyses were undertaken over a common period 1884–2008.

Months	Maximum density chronology							
	Temperature		Precipitation		Radiation		Temperature^a^	Radiation^a^
	Correlation coefficient	Response function	Correlation coefficient	Response function	Correlation coefficient	Response function	Response function	Response function
August*	**0.24**	0.02	0.11	0.12	**−0.21**	−0.15	0.06	**−0.14**
September*	−0.06	−0.12	0.02	−0.02	−0.19	−0.11	−0.04	−0.03
October*	0.06	0.02	−0.03	−0.02	0.12	0.09	−0.02	0.12
November*	0.06	0.02	**0.18**	**0.17**	0.09	0.06	0.02	0.01
December*	−0.01	−0.01	−0.01	0.06	0.02	−0.03	−0.01	−0.02
January	0.15	0.09	−0.08	−0.06	0.01	0.02	0.06	−0.01
February	−0.02	−0.05	0.08	0.04	−0.04	−0.03	−0.05	−0.03
March	0.15	0.04	0.03	0.00	0.06	0.02	0.11	−0.02
April	**0.25**	**0.15**	−0.10	−0.07	**0.28**	**0.18**	0.07	0.08
May	**0.39**	**0.19**	**−0.22**	**−0.18**	0.16	0.11	**0.21**	0.01
June	**0.18**	0.10	0.12	0.11	−0.14	−0.10	**0.12**	−0.11
July	**0.42**	**0.17**	−0.16	−0.14	**0.37**	**0.25**	**0.13**	0.07
August	**0.66**	**0.38**	−0.13	−0.11	**0.20**	**0.14**	**0.28**	0.00
September	**0.44**	**0.33**	**−0.36**	**−0.29**	**0.28**	**0.17**	**0.23**	0.10
October	−0.02	−0.07	0.11	0.10	0.15	0.08	−0.13	0.07

a Response function analysis carried out together for temperature and solar radiation data.

**Table 2:** Comparison of light ring years identified visually on the basis of dark colour of latewood visible on X-ray films with light ring years distinguished on the basis of low maximum density values. Asterisks indicate the values lower than accepted threshold of light ring years. Calendar years used during calculation of relationship between light rings and climate parameters are marked in bold.

Year	Number of series with LR calculated on the basis of MXD	Percent of MXD calculated LR	Number of series with visually distinguished LR	Percent of visually determined LR	Number of samples/sample depth
101	1	25	0	0*	4
102	1	25	0	0*	4
111	3	60	1	20	5
112	1	20*	1	20	5
117	4	40	0	0*	10
118	3	30	0	0*	10
121	3	30	0	0*	10
202	1	8*	1	8	12
257	3	33	0	0*	9
395	5	38	4	31	13
408	0	0*	1	8	13
457	3	27	0	0*	11
465	2	20*	1	10	10
536	3	60	4	80	5
744	2	25	0	0*	8
751	3	38	0	0*	8
753	2	25	0	0*	8
757	2	25	0	0*	8
762	3	33	0	0*	9
802	4	25	5	31	16
875	0	0*	1	6	16
895	5	28	2	11	18
903	7	33	4	19	21
923	7	26	3	11	27
1001	1	5*	3	14	22
1013	2	8*	4	15	26
1032	7	27	3	12	26
1040	2	7*	2	7	28
1068	5	16*	2	6	31
1084	12	32	3	8	38
1151	11	34	3	9	32
1186	18	55	8	24	33
1281	2	13*	1	6	16
1290	6	35	0	0*	17
1292	10	59	4	24	17
1299	4	19*	2	10	21
1302	8	38	2	10	21
1335	6	26	0	0*	23
1465	3	25	0	0*	12
1470	2	17*	1	8	12
1480	4	25	1	6	16
1481	4	24*	1	6	17
1675	7	33	1	5	21
1716	10	38	1	4*	26
**1805**	7	21*	4	12	33
**1814**	7	20*	2	6	35
1816	11	31	0	0*	35
**1864**	4	10*	3	7	41
**1912**	20	42	25	52	48
**1924**	9	19*	4	8	48
**1976**	13	27	10	21	48

**Table 3:** Mean monthly temperature, precipitation and radiation averaged for light ring years and remaining normal years. Student-*t* tests verify significance level of differences between those two groups of years. For calculations monthly averaged data for temperature (1780–2008 – 6 light ring years) and for solar radiation (1884–2008 – 4 light ring years), as well as monthly sums of precipitation (1800–2008 – 6 light ring years) were used.

Months	Mean temperatures and in parentheses standard deviations		Verification of the difference between light ring years and remaining years		Mean precipitations and in parentheses standard deviations		Verification of the difference between light ring years and remaining years		Mean radiations and in parentheses standard deviations		Verification of the difference between light ring years and remaining years	
	Remaining years	Light ring years	Student *t* test	Significance levels	Remaining years	Light ring years	Student *t* test	Significance levels	Remaining years	Light ring years	Student *t* test	Significance levels
January	−5.2 (2.4)	−6.4 (1.9)	1.514	*P* < 0.10	10.6 (6.2)	13.3 (8.0)	−0.816	Not significant	4.7 (1.9)	4.7 (1.3)	−0.001	Not significant
February	−4.3 (2.4)	−5.2 (3.0)	0.670	Not significant	10.5 (6.3)	9.1 (4.9)	0.709	Not significant	8.9 (3.3)	6.9 (1.3)	2.518	*P* < 0.01
March	−1.6 (2.1)	−2.1 (1.6)	0.686	Not significant	11.8 (6.3)	9.1 (5.2)	1.237	*P* < 0.10	13.5 (3.6)	15.1 (3.7)	−0.751	Not significant
April	2.3 (1.7)	1.6 (1.5)	1.154	Not significant	12.0 (5.3)	11.7 (2.5)	0.260	Not significant	16.3 (4.2)	15.0 (7.4)	0.297	Not significant
May	7.3 (1.8)	6.8 (1.4)	0.815	Not significant	13.1 (4.6)	14.7 (4.6)	−0.834	Not significant	22.1 (4.3)	23.2 (4.2)	−0.437	Not significant
June	10.3 (1.5)	9.8 (0.9)	1.511	*P* < 0.10	20.0 (5.5)	22.8 (7.1)	−0.938	Not significant	22.4 (4.3)	24.5 (4.1)	−0.865	Not significant
July	12.2 (1.4)	11.9 (0.8)	0.995	Not significant	23.2 (6.7)	22.9 (3.2)	0.217	Not significant	23.0 (4.2)	22.9 (2.4)	0.012	Not significant
August	12.0 (1.4)	9.9 (0.9)	5.650	*P* < 0.0005	21.1 (6.2)	24.1 (5.4)	−1.354	Not significant	23.4 (4.2)	17.3 (0.6)	11.533	*P* < 0.0005
September	9.1 (1.5)	7.5 (2.4)	1.655	*P* < 0.05	15.3 (6.6)	19.6 (7.1)	−1.472	Not significant	17.7 (4.0)	11.7 (8.2)	1.267	Not significant
October	4.9 (1.7)	3.9 (1.6)	1.524	*P* < 0.10	11.2 (5.9)	9.2 (5.6)	0.829	Not significant	11.4 (3.7)	9.9 (2.9)	0.881	Not significant
